# The effects of prenatal cigarette and e-cigarette exposure on infant neurobehaviour: A comparison to a control group

**DOI:** 10.1016/j.eclinm.2020.100602

**Published:** 2020-10-15

**Authors:** Suzanne Froggatt, Nadja Reissland, Judith Covey

**Affiliations:** Psychology Department, Durham University, Science site, South Road, Durham DH1 3LE, UK

## Abstract

**Background:**

Infant neurobehaviour provides an insight into the development of the central nervous system during infancy, with behavioural abnormalities highlighting a cause for concern. Research has demonstrated that prenatal exposure to cigarettes leads to deficits within neurobehavioural development, along with negative birth outcomes detrimental to subsequent development. With the growing use of e-cigarettes amongst pregnant women, this study explores how prenatal e-cigarette exposure compares to prenatal cigarette exposure.

**Methods:**

Eighty-three infants were involved in the study, either exposed prenatally to cigarettes or e-cigarettes or not exposed to either. Differences were assessed between these three groups for birth outcomes and scores on the Neonatal Behavioural Assessment Scale (NBAS) at one month of age.

**Findings:**

Both cigarette and e-cigarette exposed infants had a significantly greater number of abnormal reflexes (*p* = ·001; *p*  =  ·002). For both self-regulation and motor maturity, cigarette exposed infants performed significantly worse (*p* = ·010; *p* = ·002), with e-cigarette exposed infants having decreased motor maturity (*p* = ·036) abilities and marginally decreased for self-regulation (*p* = ·057). Birth outcomes, namely birthweight, gestation and head circumference, did not differ for e-cigarette exposed infants compared with infants who were not prenatally exposed to nicotine. Cigarette exposed infants had a significantly lower birthweight (*p* = ·021) and reduced head circumference (*p* = ·008) in comparison to non-exposed infants.

**Interpretation:**

To our knowledge, this is the first research study assessing a neurological outcome as a result of e-cigarette exposure. Findings of this have potentially important implications for public health policies regarding the safety and use of e-cigarettes throughout pregnancy.

**Funding:**

This research was funded by a doctoral training partnership scholarship via the ESRC, ES/P000762/1.

**Research in Context**

**Evidence before the study**

Scopus was searched in April 2020, with no date limit. Search terms included; “prenatal AND e-cig*” OR “e-cig* AND pregnancy” AND “infan* AND neurobehav*” OR “newborn AND neurobehav*”. No articles assessing e-cigarettes and infant neurobehaviour were found.

**Added value of the study**

This is the first study to assess any neurobehavioural responses of an infant as a result of prenatal e-cigarette exposure. The range of detrimental outcomes of prenatal cigarette exposure are well established. With public health initiatives focused on a reduction of cigarette smoking during pregnancy to 6% by 2022, despite lack of evidence regarding safety for the developing infant, e-cigarettes are used as a harm reduction method. The findings indicate that whilst birth outcomes do not appear to be affected by e-cigarette exposure, these infants do have a greater number of abnormal primitive reflexes and marginally decreased self-regulation abilities similar to prenatally cigarette exposed infants, in comparison to non-exposed infants.

**Implications of the evidence**

Further research is required to test the effects of e-cigarette use during pregnancy, alongside other forms of nicotine replacement therapy to fully explore the impact of nicotine on the infant. This study adds to the current debate regarding e-cigarette use as a method of harm reduction with possible implications for public health policy.

Alt-text: Unlabelled box

## 1 Introduction

Reducing smoking during pregnancy is a key public health priority due to a range of detrimental birth outcomes, including intrauterine growth restriction, low birth weight (<2500 g), small for gestational age, preterm delivery (<37 weeks) and reduced head circumference [1,2]. Accompanying the birth outcomes, such as low birth weight, are the neurobehavioural deficits that may occur as a result of prenatal cigarette exposure, including irritability, poor muscle tone, decreased self-regulation, increased negative affect and difficult temperament [3]. These neurobehavioural deficits have been shown to predict subsequent infant development including psychomotor, cognitive and emotional development [4]. Low birth weight in infants of mothers who smoke indicates fetal growth restriction thought to be related to Carbon Monoxide (CO) exposure affecting the oxygen carrying capacity of the fetal blood [5]. Alternatives to cigarette smoking, such as nicotine replacement therapy (NRT) and e-cigarettes are therefore considered by some to be a harm reduction method and information provided in healthcare leaflets for pregnant women state that nicotine alone is relatively harmless [6]. There is however growing concern about the increasing use of e-cigarettes and the safety of nicotine exposure for the developing fetus [7]. Therefore, assessing birth and infant outcomes in fetuses that have been exposed to e-cigarettes, will add to the debate regarding their use during pregnancy.

Although the use of e-cigarettes in pregnancy will not expose the fetus to CO, they will be exposed to nicotine which has been shown to have a negative impact on neurobehaviour. Nicotine has extensive effects on the central nervous system (CNS), with the deficits reflecting the biological and behavioural systems that are modulated through neural feedback [[8], [9], [10], [11]]. Later in childhood, exposure to nicotine has been associated to attention deficit hyperactivity disorder (ADHD) [12]. However, no research has currently been published to establish the impact of prenatal exposure to e-cigarettes may have on neurobehavioural outcomes of human infants. At present, animal studies have been the main focus emphasizing the negative result of nicotine exposure on brain development,[13] with human infant research yet to be undertaken. Primate models on the effects of nicotine exposure demonstrate that nicotine is highly selective for various brain regions with cell signaling and cell damage occurring leading to disrupted brain development. Specifically, the cognitive impairments observed are likely to be a result of proliferation and maturation in the medial prefrontal cortex of the progenitor cells leading to a decrease of glutamatergic neurons [14]. This has been shown in primates and rodents are exposed to levels of nicotine comparable to that of an adult smoker, with sufficient amount of nicotine reaching the fetal brain eliciting neurodevelopmental changes, regardless of the gestational time point nicotine is administered [13,15].

Due to the critical role of neurobehaviour in an infant's development and the lack of guidance regarding the effects of e-cigarette use during pregnancy, the present study aims to examine how prenatal exposure to e-cigarettes compares to cigarettes and to no exposure on birth outcomes (i.e. gestation at birth, birth weight and head circumference). Additionally, neurobehavioural outcomes in one-month old infants (i.e. measured using the Neonatal Behavioural Assessment Scale (NBAS) will be reported [16]. Based on current evidence it is hypothesised that there will be a significant difference in birth outcomes (i.e. shorter gestation, lower birth weight and smaller head circumference) in cigarette exposed compared with non-exposed infants, but no significant differences are expected between e-cigarette exposed infants and non-exposed infants because e-cigarette use in pregnancy is not expected to reduce the oxygen carrying capacity of fetal blood. Secondly, it is hypothesised, that due to the direct impact of nicotine on brain development, e-cigarette exposed infants will demonstrate a similar pattern of neurobehavioural deficits to cigarette exposed infants. This is the first study assessing the neurobehavioural outcomes of the new-born as a result of nicotine exposure via e-cigarette use.

## 2 Methods

The report is written in accordance to the STROBE guidelines [17]. Ethical approval was granted by Durham University and mothers provided informed consent before any assessment was conducted.

This case-control study includes 83 white British infants who were assessed in their home at one time point at approximately one month of age (*m*  =  32.6 days, S.D. = 5.33) using the NBAS [16]. These infants were part of a larger study assessing fetal and infant behavioural development in relation to nicotine exposure conducted in collaboration with The James Cook University Hospital, Middlesbrough, UK. Eligibility criteria for inclusion was the infant was born at term (>37 weeks), healthy and no NICU admission, no prenatal alcohol consumption and no prescription or recreational drug use. Women using alternative methods of NRT such as patches, gum or inhaler were not eligible for this study due to the interest in e-cigarettes as a harm reduction method.

The e-cigarette use and cigarette smoking behavior of the mother was obtained at 32 weeks gestation due to the known effects of nicotine exposure on the fetal brain leading to behavioural differences in the early infancy period [10]. Smoking status was self-reported with a CO breath test to confirm nicotine groupings (see Table 1). All mothers were assessed using the Bedfont Smokerlyser breath test, with scores >3 parts per million (ppm) for CO indicative of mothers who smoked. This measure was used to confirm maternal self-report of smoking status. For e-cigarette users, milligrams of nicotine stated on the product's packaging was self-reported. Two prenatal e-cigarette users reverted back to cigarette use following the birth of their infant, but due to prenatal exposure, these infants remained in the prenatal e-cigarette exposure group. The demographic information for each group is shown in Table 1.

**Table 1:** Demographic information.

Nicotine group	Mean CO reading (% of CO in maternal blood)	Number of infants	Gender Male/Female	Number of households with additional cigarette smokers	Mean years of maternal cigarette use prior to conception	Number of primiparous mothers	Highest educational qualification
Non-exposed	0·97	44	23/21	3	0·34	21	None: 0 GCSE: 9 College/A-levels: 9 Degree: 18 Masters: 8
Cigarette exposed (1–20 per day)	2·74	29	15/14	10	11·2	8	None: 9 GCSE: 14 College/A-levels: 4 Degree: 2 Masters: 0
E-cigarette exposed (3–16 mg in the product)	0·95	10	1/9	2	4·2	7	None: 0 GCSE: 5 College/A-levels: 5 Degree: 0 Masters: 0

Birth outcomes for each infant were received from the hospital or recorded at the one month follow up. Given the known association between maternal mental health to both fetal and infant outcomes,[18] mothers completed a range of questionnaires assessing perceived stress,[19] depression and anxiety as measured by the Hospital Anxiety and Depression Scale [20] at the 32 week ultrasound scan. A postnatal attachment questionnaire was completed at the one month follow up [21]. Alongside maternal age and additional household smokers, these factors were controlled for in the analysis where appropriate.

For measures of orientation, motor maturity, range of states, regulation and automatic stability, the NBAS scores infants on a Likert scale from 1 to 9 [16] and recoded following the method outlined by Lester (1984; as cited in Brazelton & Nugent, 1995). The reflexes were tested for the number of abnormal reflexes [22]. Seventeen reflexes were assessed as outlined by the NBAS including; Plantar, Babinski, ankle clonus, rooting, glabella, passive leg tone, passive arm tone, palmer grasp, placing, standing, stepping, crawling, incurvation, tonic deviation, nystagmus, TNR and Moro. These reflexes were rated at the time of the assessment between 0 and 3. For ankle clonus, nystagmus and TNR, scores of 3 are considered abnormal. For all other reflexes, a score of 2 is normal and scores of 0, 1 or 3 are considered abnormal. Normal reflexes are co-ordinated, strong and modulated responses, anything other is considered abnormal such as weak reflexes or obligatory reflexes with little relaxation following the end of the reflex [16].

### Data analysis

ANOVAs were conducted to assess group differences for birth outcomes (gestation, birthweight and head circumference) and NBAS outcomes (reflexes, regulation, motor maturity, orientation, range of states and automatic stability). Seven potential covariates (maternal age, infant sex, primiparity, additional household smokers, stress, depression and anxiety) were correlated with each outcome measure to assess suitability for inclusion in an ANCOVA. Covariates which significantly correlated with the outcomes were included in the ANCOVA.

We also correlated the self-reported mg of nicotine (for the e-cigarette group) and the number of years the mother smoked prior to conception (all exposure groups) with NBAS outcomes. However, given the data is not independent of exposure group, significant correlations could not be included in the ANCOVA.

Series means estimates were used for missing data. Bootstrap methods were employed due to the small sample and likely variation within the population, 1000 resamplings were performed. Analysis was conducted using the Statistical Package for the Social Sciences version 26 (SPSS).

### Role of the funding source

The funding source had no involvement in the study design, data collection, data analysis, interpretation, report writing or decision to submit the paper for publication.

## Results

The aims of the study were to assess whether birth outcomes and neurobehavioural outcomes differed between prenatal non-exposed, cigarette exposed and e-cigarette exposed infants.

As shown in Table 2, there were significant differences in maternal age between the groups, *F*(2,82) = 8·263, *p* = ·001, η^2^ = 0.171. Mothers who did not smoke during pregnancy were significantly older in comparison to smokers (*p* = ·004, *d*  =  0·680) and e-cigarette users (*p* = ·001, *d*  =  1·253). None of the other covariates were significantly different between the groups. The correlations between the covariates and the birth outcomes and NBAS measures are shown in Table 3. Only covariates that significantly correlated with the outcomes were included in the ANCOVA.

**Table 2:** Means and standard deviations for birth outcomes, maternal characteristics and NBAS outcomes split by nicotine group.

	Mean Non-exposed (a)	Standard deviation	Mean Cigarette exposed^(b)^	Standard deviation	Mean E-cigarette exposed ^(c)^	Standard deviation
Maternal age (years)*a-ba-c	28·84	4·86	25·52	4·911	22·60	5·52
Stress	10·64	6·36	13·14	6·84	15·40	4·37
Depression	2·86	2·59	5·21	3·29	4·50	2·71
Anxiety	4·55	3·02	6·41	3·67	5·50	2·75
Attachment	72·104	3·979	72·942	3·062	71·026	3·952
Gestation (weeks)	39·178	1·36	39·11	1·26	39·98	·77
Birthweight (grams)*a-b	3451·92	596·69	3098·37	434·89	3477·11	257·91
Head circumference (cm)*a-b	34·75	1·48	33·63	1·45	34·38	·89
Apgar 1 min	8·833	·618	8·935	·428	8·841	·319
apgar 5 min	9·435	·455	9·592	·473	9·178	·576
labor length (minutes)	287·699	192·719	311·827	298·391	250·375	178·959
Reflexes*a-ba-c	2·11	1·72	4·59	2·18	5·60	2·503
Orientation	6·18	1·38	5·83	·94	5·63	1·60
Motor maturity*a-b	5·97	·57	5·39	·82	5·48	·755
Range of states	3·70	·97	3·55	·95	3·80	1·01
Regulation*a-b	4·88	1·22	4·20	·84	3·80	1·76
Automatic stability	6·97	1·18	7·08	1·08	7·21	·83

a-b significant posthoc between non-exposed and cigarette exposed.

a-c significant posthoc between non-exposed and e-cigarette exposed.

b-c significant posthoc between cigarette exposed and e-cigarette exposed.

⁎ Significant main effect, *p*<.05.

**Table 3:** Correlations (with p-values) between maternal and infant characteristics and birth outcomes and NBAS outcomes.

	Maternal age	Stress 32	Anxiety 32	Depression 32	Attachment Postnatal	Additional Smokers	Number of years smoked prior to conception	Infant sex	Primiparity
Gestation	−0·116 (0·296)	−0·071 (0·526)	−0·097 (0·384)	−0·109 (0·327)	·120 (0·320)	·080 (0·473)	−0·038 (0·736)	·129 (0·282)	·095 (0·395)
Birthweight	−0·089 (0·423)	−0·076 (0·266)	−0·123 (0·266)	−0·020 (0·857)	−0·188 (0·117)	−0·012 (0·916)	−0·292 (0·007)*	·118 (0·324)	·022 (0·843)
Head circumference	−0·102 (0·360)	−0·037 (0·737)	−0·093 (0·405)	−0·037 (0·742)	−0·052 (0·667)	−0·132 (0·234)	−0·292 (0·007)*	·071 (0·551)	−0·010 (0·927)
Reflex	−0·204 (0·064)	·118 (0·288)	·147 (0·184)	·263 (0·016)*	−0·114 (0·345)	·157 (0·157)	·432 (<0·001)*	−0·184 (0·121)	−0·175 (0·113)
Motor maturity	·218 (0·047)*	−0·033 (0·768)	−0·139 (0·209)	−0·253 (0·021)*	−0·232 (0·051)	−0·033 (0·770)	−0·232 (0·035)*	−0·014 (0·905)	·125 (0·254)
Regulation	·022 (0·844)	−0·097 (0·387)	−0·095 (0·394)	−0·114 (0·306)	−0·020 (0·868)	·001 (0·991)	−0·226 (0·042)*	·016 (0·891)	−0·020 (0·861)
Orientation	−0·062 (0·584)	·017 (0·880)	−0·032 (0·775)	−0·139 (0·217)	·011 (0·929)	·004 (0·971)	−0·179 (0·111)	·185 (0·126)	·150 (0·182)
Range states	−0·083 (0·457)	−0·053 (0·634)	·026 (0·813)	·056 (0·616)	−0·090 (0·868)	−0·079 (0·479)	−0·075 (0·500)	−0·010 (0·930)	·177 (0·110)
Automatic stability	−0·116 (0·296)	·034 (0·763)	·024 (0·831)	−0·034 (0·760)	−0·231 (0·053)	−0·008 (0·940)	·034 (0·758)	−0·058 (0·626)	·163 (0·141

The Perceived Stress Scale was administered prenatally at the mother's 32-week hospital ultrasound appointment.

The Hospital Anxiety and Depression Scale was administered prenatally at the mother's 32-week hospital ultrasound appointment.

As this measure is not independent of the IV (exposure group), significant correlations could not be included in the ANCOVA.

⁎ *p*<.05.

Regarding birth outcomes, no significant differences for gestation at birth between the three exposure groups were observed, *F*(2,82)  =  1·652, *p* = ·198, η^2^ = 0·040. Significant differences were observed for birthweight, *F*(2,82)  =  4·192, *p* = ·019, η^2^ = 0·095. Pairwise comparisons applying the Bonferroni correction confirmed that cigarette exposed infants had a significantly lower birthweight in comparison to non-exposed infants (*p* = ·021, *d*  =  0·656), but differences in birthweight for e-cigarette exposed compared to non-exposed and cigarette infants was not significant (*p*  =  1, *d*  =  0·030; *p* = ·188, *d*  =  0·893). None of the covariates were significantly correlated with birthweight (see Table 3). Therefore, no ANCOVA was conducted.

There were also significant differences between the exposure groups in head circumference, *F*(2,82) = 4·771, *p* = ·011, η^2^ = 0·107. Cigarette exposed infants had a significantly reduced head circumference in comparison to non-exposed infants (*p* = ·008, *d*  =  0·763), with e-cigarette exposed infants not differing to non-exposed infants (*p*  =  1, *d*  =  0·242) or cigarette exposed infants (*p* = ·525, *d*  =  0·533). No covariate correlated with head circumference (see Table 3), therefore no ANCOVA was conducted.

Significant differences were observed across the nicotine groups for reflexes *F*(2,82)  =  20·338, *p*<·001, η^2^ = 0·338, motor maturity, *F*(2,82)  =  6·769, *p* = ·002, η^2^ = 0·145, and regulation *F*(2,82)  =  4·877, *p* = ·010, η^2^ = 0·110. There were no significant differences observed for measures of orientation (*p* = ·340, η^2^ = 0·027), range of states (*p* = ·725, η^2^ = 0·008) and automatic stability (*p* = ·798, η^2^ = 0·006). There were significant correlations between number of years smoked prior to conception and reflexes (*r*  =  0·432, *p* = <0·001), motor maturity (*r* = −0·232, *p* = .035) and regulation (*r* = −0·226, *p* = ·758). In addition, there was a significant correlation between mg of nicotine in the e-cigarette exposure group and motor maturity (*r* = −0·349, *p* = ·001), however no other NBAS outcome measures were significantly associated with mg of nicotine.

Pairwise comparisons applying the Bonferroni correction for reflexes indicate significant differences between infants not exposed and exposed to cigarettes (*p* = ·001, *d*  =  1·263) and e-cigarettes (*p* = ·002, *d*  =  1·625). There were no significant differences found between cigarette exposed and e-cigarette exposed infants (*p* = ·236, *d*  =  0·287). Similarly, when adjusting for maternal depression (see Table 3), significant differences were observed across the three nicotine groups for reflexes *F*(2,82)  =  16·479, *p*<·001, η^2^ = 0·294. Assessing the pairwise comparison for the NBAS outcomes accounting for maternal depression using the Bonferroni correction, significant differences were found between non-exposed and cigarette exposed (*p* = ·001, *d*  =  1·263) and e-cigarette exposed infants (*p* = ·001, *d*  =  1·625).

Similarly, for motor maturity, pairwise comparisons with the Bonferroni correction indicate significant differences between non-exposed and those exposed to cigarettes (*p* = ·002, *d*  =  0·821) and between non-exposed and e-cigarette exposed infants (*p* = ·036, *d*  =  0·732). There were no significant differences between e-cigarette and cigarette exposed infants (*p* = .745, *d*  =  0·103). When controlling for maternal age and maternal depression, this effect becomes marginal, *F*(2,82)  =  2·941, *p* = ·059, η^2^ = 0·070.

For regulation, pairwise comparisons with the Bonferroni correction indicate significant differences between non-exposed and those exposed to cigarettes (*p* = ·010, *d*  =  0·649). There were no significant differences between non-exposed and e-cigarette exposed infants (*p* = ·057, *d*  =  0·713) and between cigarette exposed and e-cigarette exposed infants (*p* = ·454, *d*  =  0·358). No covariates were significantly correlated to regulation (see Table 3), therefore ANCOVA was not conducted.

## Discussion

It was hypothesised that there would be a significant difference in birth outcomes (birthweight, gestation at birth and head circumference) between cigarette exposed and non-exposed infants, but no significant difference between e-cigarette exposed and non-exposed infants. Secondly, it was hypothesised that e-cigarette exposed infants will demonstrate similar neurobehavioural outcomes to cigarette exposed infants, compared to non-exposed infants. These hypotheses received partial support.

The results regarding the birth outcomes indicate that, in contrast to previous research [23,24], there is no significant difference between cigarette exposed and non-exposed infants for gestation at birth. The majority of research assessing prenatal cigarette exposure and gestation at birth focuses on the greater risk of preterm delivery before <37 weeks gestation. However, in the present study, infants were only included if they were born at least 37 weeks gestation, due to the associated complications with preterm delivery such as poorer physiological health and developmental immaturity [25]. This could explain why we did not find a difference between cigarette and non-exposed groups. Nevertheless, as predicted there are significant differences regarding birthweight and head circumference between these two groups. For e-cigarette exposed infants, no significant differences were observed in comparison to non-exposed infants for gestation, birthweight or head circumference, in line with previous findings and our predictions [26]. In this particular sample, there is no evidence suggesting birth outcomes are affected as a result of e-cigarette exposure.

Given that infants prenatally exposed to e-cigarettes did not experience the same birth outcomes as cigarette exposed, but were similar to non-exposed infants, it could indicate a likely culprit for these negative outcomes is CO exposure, It is well established that CO exposure is associated with low birth weight [5,27]. This is due to CO binding to hemoglobin reducing blood flow and subsequently leading to growth restriction [10]. Based on the current findings, when CO is removed, through use of an e-cigarette, low birth weight appears to be no longer concerning, however, further exploration on larger samples is needed to add further support.

In relation to NBAS outcomes, the results indicate that motor maturity, self-regulation and reflexes are different across exposure groups. Interestingly, these measures were also correlated to number of years the mothers smoked prior to conception. The longer the mother smoked, the worse the infants’ regulation and motor maturity, and these infants would also demonstrate a greater number of abnormal reflexes. Epigenetic research argues that smoking can have a cumulative effect, with the month prior to conception being a critical time point for early placental development, with altered development leading to changes in brain structure and function [28].

The findings indicated that both cigarette exposed and e-cigarette exposed infants demonstrate a decrease in motor maturity when compared to non-exposed infants. However, in contrast to previous literature [3], when the maternal age and maternal depression were controlled for, the effect smoking has on motor maturity was no longer significant. The differences between the groups might partly reside in the fact that the non-smokers in our sample were older and reported fewer depressive symptoms, although not significant, in comparison to the mothers using e-cigarettes or smoking. Interestingly, mg of nicotine for the e-cigarette exposed infants correlated with their motor maturity score, indicating that the higher the mg of nicotine, the lower they score on motor maturity.

In regard to self-regulation, cigarette exposed infants displayed decreased abilities in comparison to non-exposed infants, which is consistent with previous research [3]. Although the difference between non-exposed and e-cigarette exposed infants was not significant, this result was approaching significance with a large effect size. Measures of self-regulation include self-relaxation of the infant when held, how consolable the infant is following a period of crying, self-quieting abilities and hand-to-mouth movements [16]. Infants who demonstrate decreased self-regulation abilities are often more irritable and need external consoling. Regulation is important for subsequent infant psychomotor and emotional development. In addition, early regulation abilities predict development at 4 and 12 months and in turn predict intellectual development at 6 years of age [4]. Because of potential long-term consequences associated with decreased self-regulation abilities, and due to the large effect size, this warrants further exploration.

The novel findings reported here demonstrate the negative effect e-cigarettes have on reflexes. When controlling for maternal depression, a large effect size was shown between non-exposed and e-cigarette exposed infants, with the latter demonstrating more abnormal reflexes. The results between non-exposed and cigarette exposed infants are supported by previous research [3]. It is likely that these results are generalisable to the population, given the large effect size. Given that reflexes are related to both cigarettes and e-cigarette exposure, this suggests that nicotine consumption in pregnancy regardless of delivery method is a potential cause for concern.

Primitive reflexes have a developmental role allowing the infant to interact with their environment in a basic way, essential for new born survival and preparing the infant for voluntary movements [29,30]. These reflexes are automatic involuntary patterns of movement that are mediated by the brainstem [31]. They support the development of natural movement patterns allowing the infant to reach early voluntary motor milestones such as grasping, rolling and crawling [29]. They gradually reduce when the infant is between 4 and 6 months of age and occurs once the CNS matures with movements becoming voluntary, with retained reflexes a cause for concern. The CNS maturation leads to a transition of control of movements from brainstem responses, to cortically controlled responses [32]. As primitive reflexes are controlled by the CNS, mediated by the brainstem [32]. it is likely that exposure group differences are a result of the widespread effects of nicotine activating nicotinic acetylcholine receptors (nAChRs) across the CNS [33].

These results may have occurred due to exposure to nicotine prenatally. The fetal brain is susceptible to damage and the vulnerability is dependent upon whether a toxin can penetrate the fetal CNS [34]. The developing brain is protected from a range of neurotoxins, however, nicotine readily crosses the syncytium, targeting specific neurotransmitters, causing an accumulation of nicotine in fetal tissue, ultimately resulting in impaired fetal brain development [35]. NAChRs that are widespread throughout the CNS controlling cell replication and differentiation [13,33]. Rodent studies indicate brain growth restriction, fetal hypoxia and brain development are negatively impacted by prenatal nicotine exposure as a result of nAChRs expression [33]. However, a key concern of reflecting on rodent studies to provide an indication of the impact of nicotine is that in comparison to human infants, rodents have a longer period of postnatal CNS maturation, therefore comparison is difficult [34]. However, primate studies do not pose such problems, yet have found similar results. In primates, nicotine exposure leads to cell damage and cell signaling disruptions leading to changes within brain development [13]. Whilst animal studies indicate the brain changes as a result of prenatal nicotine exposure, they are unable to provide evidence of ‘real-life’ application effects, such as neurobehavioural implications. Therefore, in order to provide evidence for policy change, research should focus on the impact on human infants.

A concern is that e-cigarettes are termed a harm reduction method for use in pregnancy [7]. However, the present findings indicate that there could be harm associated with e-cigarette use and therefore the ultimate aim must be to stop smoking, without the use of e-cigarettes. Indeed, caution should probably be applied to all NRT products. Given the predictive nature of newborn assessments [4], in particular the NBAS, the notion that nicotine by itself is relatively harmless, is a concept that needs to be further questioned and further investigated.

Further research is vital in order to establish the effects of nicotine on postnatal neurological outcomes, including a biological element. It is difficult to quantify how much of an e-cigarette is used on a daily basis and in this study self-report was relied on to measure mg of nicotine in the e-cigarette product. This is in comparison to daily self-reported use of cigarettes which may be easier to quantify. Therefore, a more objective measure of nicotine exposure, via cotinine, would aid further development of such research. Cotinine is a metabolite of nicotine and can be measured in both the smoker and those exposed to second hand smoke [36]. Whilst measuring cotinine can provide further evidence to support the effects of nicotine on infant neurobehavioural outcomes, it is important to note that e-cigarettes contain a variety of other toxic compounds. For example one study identified metals present in the e-liquid vapor such as cadmium, chromium, lead, manganese and nickel which could also be producing carcinogenic effects [37]. Nonetheless, given that this research has demonstrated that nicotine exposure through e-cigarette use is associated with a significantly greater number of abnormal reflexes, future research needs to explore the risks associated with NRT, such as patches and inhalers for use in pregnancy.

An additional limitation of the research, as with all epidemiological research, is the potential impact of unmeasured possible confounding factors. For example, in this study, socioeconomic status (SES) was not assessed. And although research suggests that SES can influence child development through its effects on how parents interact with their children, there is little evidence that SES is directly associated with infant outcomes [9,38].

This is the first study assessing neurobehavioural outcomes associated with prenatal nicotine exposure through cigarettes or e-cigarettes at one month old. Overall, results indicate that birthweight, gestation and head circumference measurements do not differ between prenatal e-cigarette exposure and no exposure. Importantly, regardless of prenatal nicotine exposure (cigarettes or e-cigarettes), this research found a significantly greater number of abnormal primitive reflexes, alongside marginally decreased self-regulation abilities compared with non-exposed infants. These findings have important implications for policy guidelines regarding the use and safety of e-cigarettes during pregnancy as a method of harm reduction.

## Declaration of Competing Interest

The authors declare there is no conflict of interest.

## Appendix Supplementary materials

Image, application 1
